# Evaluating the Total Healthcare Cost of Inappropriately Ordered Thyroid Ultrasounds

**DOI:** 10.1210/jendso/bvaf143

**Published:** 2025-09-02

**Authors:** David Toro-Tobon, Viengneesee Thao, Bijan J Borah, Cristian Soto Jacome, Felipe Larios, Kerly Guevara, Luis Vilatuna Andrango, Ana Cristina Proano, Jungwei W Fan, Ricardo Loor-Torres, Misk Al Zahidy, Esteban Cabezas, Yuqi Wu, Megan E Branda, Naykky Singh Ospina, Juan P Brito

**Affiliations:** Division of Endocrinology, Diabetes, Metabolism, and Nutrition, Mayo Clinic, Rochester, MN 55905, USA; Knowledge and Evaluation Research Unit, Division of Endocrinology, Diabetes, Metabolism, and Nutrition, Department of Medicine, Mayo Clinic, Rochester, MN 55905, USA; Robert D. and Patricia E. Kern Center for the Science of Health Care Delivery, Mayo Clinic, Rochester, MN 55905, USA; Robert D. and Patricia E. Kern Center for the Science of Health Care Delivery, Mayo Clinic, Rochester, MN 55905, USA; Knowledge and Evaluation Research Unit, Division of Endocrinology, Diabetes, Metabolism, and Nutrition, Department of Medicine, Mayo Clinic, Rochester, MN 55905, USA; Department of Medicine, Norwalk Hospital, Norwalk, CT 06856, USA; Robert Larner College of Medicine, University of Vermont, Burlington, VT 05405, USA; Knowledge and Evaluation Research Unit, Division of Endocrinology, Diabetes, Metabolism, and Nutrition, Department of Medicine, Mayo Clinic, Rochester, MN 55905, USA; Knowledge and Evaluation Research Unit, Division of Endocrinology, Diabetes, Metabolism, and Nutrition, Department of Medicine, Mayo Clinic, Rochester, MN 55905, USA; Knowledge and Evaluation Research Unit, Division of Endocrinology, Diabetes, Metabolism, and Nutrition, Department of Medicine, Mayo Clinic, Rochester, MN 55905, USA; Knowledge and Evaluation Research Unit, Division of Endocrinology, Diabetes, Metabolism, and Nutrition, Department of Medicine, Mayo Clinic, Rochester, MN 55905, USA; Department of Artificial Intelligence and Informatics, Mayo Clinic, Rochester, MN 55905, USA; Knowledge and Evaluation Research Unit, Division of Endocrinology, Diabetes, Metabolism, and Nutrition, Department of Medicine, Mayo Clinic, Rochester, MN 55905, USA; Knowledge and Evaluation Research Unit, Division of Endocrinology, Diabetes, Metabolism, and Nutrition, Department of Medicine, Mayo Clinic, Rochester, MN 55905, USA; Knowledge and Evaluation Research Unit, Division of Endocrinology, Diabetes, Metabolism, and Nutrition, Department of Medicine, Mayo Clinic, Rochester, MN 55905, USA; Knowledge and Evaluation Research Unit, Division of Endocrinology, Diabetes, Metabolism, and Nutrition, Department of Medicine, Mayo Clinic, Rochester, MN 55905, USA; Department of Artificial Intelligence and Informatics, Mayo Clinic, Rochester, MN 55905, USA; Division of Biomedical Statistics and Informatics, Department of Health Sciences Research, Mayo Clinic, Rochester, MN 55905, USA; Division of Endocrinology, Department of Medicine, University of Florida, Gainesville, FL 32610, USA; Division of Endocrinology, Diabetes, Metabolism, and Nutrition, Mayo Clinic, Rochester, MN 55905, USA; Knowledge and Evaluation Research Unit, Division of Endocrinology, Diabetes, Metabolism, and Nutrition, Department of Medicine, Mayo Clinic, Rochester, MN 55905, USA

**Keywords:** thyroid ultrasound, healthcare costs, low-value care, overdiagnosis, cascade of care, natural language processing

## Abstract

**Purpose:**

Overuse of thyroid ultrasound (TUS) has contributed to rising thyroid cancer diagnoses and is projected to increase US healthcare costs from $1.5 billion to $3.5 billion by 2030. This study evaluated the healthcare cost of inappropriately ordered TUS in a national multicenter academic system.

**Methods:**

This is a secondary cost analysis of a retrospective cohort study across 4 Mayo Clinic sites (Rochester, MN; Jacksonville, FL; Scottsdale, AZ; and the Midwest Mayo Clinic Health System). Adult patients (≥18 years) undergoing their first TUS between January 1, 2017, and December 31, 2021, with at least 1 year of follow-up were included. TUS indications were classified as appropriate or inappropriate using a guideline-based natural language processing algorithm. The primary outcome was a comparison of adjusted 1-year all-cause healthcare costs. A secondary analysis calculated the direct procedural costs of the inappropriate TUS cascade.

**Results:**

Among 6984 patients (mean age 56 [SD 16.4]; 76.2% female; 90.9% White), 546 (7.8%) underwent TUS for inappropriate indications. These patients were younger (mean age 53.0 vs 56.3 years, *P* < .0001) but otherwise demographically similar. Adjusted total healthcare costs over 90 days and 1 year were comparable: $4842 vs $5794 and $13 748 vs $14 257 for inappropriate vs appropriate TUS, respectively. The inappropriate TUS cascade, including an estimated 56 subsequent biopsies and 22 thyroidectomies, resulted in a minimum of $576 134 in direct procedural costs.

**Conclusion:**

While adjusted total costs were similar, inappropriate TUS represents potentially avoidable spending and remains a viable target for cost-reduction strategies. Reducing low-value imaging remains a critical target for cost-saving interventions.

Over the past 3 decades, the incidence of thyroid cancer in the United States has increased significantly, largely driven by the detection of small papillary thyroid cancers (≤1.5 cm) through imaging advancements [1, 2]. Despite an estimated 15 to 20 million undiagnosed cases, mortality rates remain low, highlighting a growing concern over overdiagnosis—where indolent cancers unlikely to impact health are detected and treated unnecessarily. This phenomenon can lead to invasive procedures, lifelong thyroid hormone replacement, emotional distress, reduced quality of life, and financial hardship, including bankruptcy in extreme cases [3]. Moreover, the annual healthcare costs associated with thyroid cancer are projected to rise from $1.5 billion to $3.5 billion by 2030 [4, 5].

Thyroid ultrasound (TUS) plays a central role in this overdiagnosis epidemic [6]. Between 2002 and 2012, TUS utilization among Medicare patients surged from 200 to 1500 exams per 100 000 people annually—a 20% year-over-year increase. Although 80% to 90% of TUS orders are guideline-concordant (eg, for evaluating nodule-related symptoms or incidental findings), as many as 10% to 40% are deemed inappropriate [7, 8]. Common inappropriate indications include screening asymptomatic individuals, investigating hypothyroidism, and responding to patient requests—contributing to unnecessary diagnoses and escalating healthcare expenditures.

In this study, we evaluate the downstream economic impact of inappropriately ordered TUS across a large, multisite academic healthcare system. We assess differences in all-cause healthcare costs, reflecting total healthcare expenditures rather than thyroid-specific or procedure-related costs. By identifying differences in healthcare utilization and associated costs between appropriate and inappropriate TUS, we aim to highlight opportunities for cost containment and promote evidence-based practice.

## Materials and Methods

### Study Design and Setting

This study is a secondary cost analysis of a cohort from a previously published study that described the factors and clinical outcomes associated with inappropriate TUS [9]. We conducted a retrospective cohort study including all adult patients (>18 years) who underwent an initial TUS between January 1, 2017, and December 31, 2021, at 1 of 4 Mayo Clinic sites: Rochester, Minnesota; Jacksonville, Florida; Scottsdale, Arizona; or the Mayo Clinic Healthcare System in the Midwest. Patients treated in Minnesota were included only if they had provided research authorization. All patients were required to have at least 1 year of follow-up after the index TUS. The final cohort for this cost analysis (n = 6984) is a subset of the parent study cohort (n = 11 442) for whom complete 1-year cost data were available [9]. Relevant clinical and demographic variables, including age, sex, comorbidities, and social determinants of health, were extracted from the electronic health record. This study was approved by the Mayo Clinic Institutional Review Board (24-006512).

### Classification of TUS Appropriateness

To assess TUS appropriateness, we applied a previously developed natural language processing (NLP) algorithm with a sensitivity 0.96, specificity 0.80, accuracy 0.96, and F1 score of 0.97 [10]. This algorithm automatically analyzes unstructured clinical documentation to classify TUS indications as appropriate or inappropriate based on accepted clinical indications. A TUS was deemed appropriate if clinical documentation reflected 1 or more of the following: evaluation of thyroid-related symptoms suggestive of a mass or nodularity; follow-up of an incidental thyroid abnormality discovered on unrelated imaging; screening in individuals with a known personal or family history of hereditary thyroid cancer syndromes; further investigation of thyroid enlargement or nodules noted on physical examination; or evaluation as part of preoperative planning for hyperparathyroidism (Supplement 1) [11].

### Outcomes and Cost Estimation

The primary outcome was the comparison of total healthcare costs associated with inappropriate vs appropriate TUS. We identified downstream healthcare utilization occurring within 90 days and 1 year following the index TUS, including additional thyroid ultrasounds, fine-needle aspiration biopsies, thyroid surgeries, thyroid hormone replacement therapy, and thyroid-related outpatient consultations.

Cost data were obtained from the Mayo Clinic's standardized cost data warehouse, which integrates Medicare reimbursement rates for professional services and hospital cost-to-charge ratios for institutional services [12]. We reported both unadjusted and adjusted total expenditures over 90-day and 1-year periods. Costs were categorized using Current Procedural Terminology and Uniform Billing revenue codes into standardized cost buckets: anesthesia, durable medical equipment, room and board, supplies, procedures, therapeutic radiology, imaging, evaluation and management, pharmacy, treatments, tests, and other services (Supplement 2) [13].

In addition to the primary all-cause cost analysis, we conducted a secondary direct cost analysis to estimate the procedural costs directly attributable to inappropriate TUS. To project the number of downstream procedures, we applied the rates of fine-needle aspiration (10.3%), partial thyroidectomy (1.6%), and total thyroidectomy (2.3%) observed in the inappropriate TUS group from the parent cohort [9] to our analytic sample of 546 patients. Per-procedure cost estimates were derived from published payer-negotiated prices, cost-effectiveness models, and real-world reimbursement data to better reflect total procedural costs [14-16]. The total estimated direct procedural cost was calculated by multiplying the projected number of each procedure by its corresponding cost. This approach provides a conservative lower-bound estimate, as it excludes additional related expenses such as laboratory tests, medications, and follow-up consultations.

### Statistical Analysis

Adjusted costs were estimated using a generalized linear model with a gamma distribution and log link function, accounting for baseline patient characteristics [17]. This approach was chosen due to the right-skewed nature of healthcare cost data. In addition, we estimated potential healthcare savings by projecting cost reductions achievable through a decrease in inappropriate TUS utilization.

## Results

Our sample consisted of a total of 6984 patients (mean age 56 [SD 16.4], 76.2% female [n = 5322], 90.9% White [n = 6346]) who underwent TUS between January 2017 and June 2023. Of these, 546 (7.8%) were classified as having received an inappropriate TUS (Table 1). Patients in the inappropriate TUS group were significantly younger than those in the appropriate TUS group (mean age 53.0 vs 56.3 years, *P* < .0001), but there were no significant differences in sex, race, body mass index, or Charlson comorbidity scores. However, patients with inappropriate TUS were more likely to have comorbid thyroid-related conditions, including hypothyroidism (44.0% vs 22.4%, *P* < .0001), hyperthyroidism (28.9% vs 8.3%, *P* < .0001), Hashimoto's thyroiditis (6.4% vs 2.8%, *P* < .0001), and Graves' disease (3.1% vs 0.9%, *P* < .0001). Inappropriate TUS orders were more frequently placed by family medicine providers (59.5% vs 51.6%, *P* = .0009) and among privately insured patients (64.1% vs 56.1%, *P* = .0059).

**Table 1.** Cohort characteristics

	Thyroid ultrasound			
	Appropriate (n = 6438)	Inappropriate (n = 546)	Total (n = 6984)	*P*-value
Age				<.0001*^a^*
Mean (SD)	56.3 (16.3)	53.0 (17.1)	56.0 (16.4)	
Sex, n (%)				.2920*^b^*
Female	4916 (76.4)	406 (74.4)	5322 (76.2)	
Male	1522 (23.6)	140 (25.6)	1662 (23.8)	
Race, n (%)				.1764*^b^*
White	5858 (91.0)	488 (89.4)	6346 (90.9)	
Black	248 (3.9)	19 (3.5)	267 (3.8)	
Native Hawaiian/other Pacific Islander	194 (3.0)	23 (4.2)	217 (3.1)	
American Indian/Alaska Native	14 (0.2)	2 (0.4)	16 (0.2)	
Asian	5 (0.1)	2 (0.4)	7 (0.1)	
Missing	119 (1.8)	12 (2.2)	131 (1.9)	
Ethnicity, n (%)				.4375*^b^*
Not Hispanic	6108 (94.9)	519 (95.1)	6627 (94.9)	
Hispanic	230 (3.6)	22 (4.0)	252 (3.6)	
Missing	100 (1.6)	5 (0.9)	105 (1.5)	
Body mass index				.0722*^a^*
Mean (SD)	30.2 (7.1)	29.9 (7.5)	30.2 (7.2)	
Charlson: sum of diseases				.1764*^a^*
Mean (SD)	0.8 (1.2)	0.7 (1.2)	0.8 (1.2)	
Comorbid conditions of interest				
Hypothyroidism	1440 (22.4)	240 (44.0)	1680 (24.1)	<.0001*^b^*
Hyperthyroidism	532 (8.3)	158 (28.9)	690 (9.9)	<.0001*^b^*
Thyroiditis Hashimoto	181 (2.8)	35 (6.4)	216 (3.1)	<.0001*^b^*
Graves' disease	60 (0.9)	17 (3.1)	77 (1.1)	<.0001*^b^*
TUS ordering site,*^c^* n (%)				<.0001*^b^*
Site A	3036 (47.2)	227 (41.6)	3263 (46.7)	
Site B	1683 (26.1)	81 (14.8)	1764 (25.3)	
Site C	931 (14.5)	154 (28.2)	1085 (15.5)	
Site D	785 (12.2)	84 (15.4)	869 (12.4)	
Site E	3 (0.0)	0 (0.0)	3 (0.0)	
TUS ordering clinician specialty, n (%)				.0009*^b^*
Family medicine	3321 (51.6)	325 (59.5)	3646 (52.2)	
Internal medicine	1277 (19.8)	92 (16.8)	1369 (19.6)	
Endocrinology	679 (10.5)	48 (8.8)	727 (10.4)	
Oncology/hematology	207 (3.2)	24 (4.4)	231 (3.3)	
Other	954 (14.8)	57 (10.4)	1011 (14.5)	
Insurance, n (%)				.0059*^b^*
Private	3609 (56.1)	350 (64.1)	3959 (56.7)	
Medicare	2001 (31.1)	130 (23.8)	2131 (30.5)	
Medicaid	529 (8.2)	41 (7.5)	570 (8.2)	
Other government program	89 (1.4)	5 (0.9)	94 (1.3)	
Missing	205 (3.2)	20 (3.7)	225 (3.2)	
Welfare	5 (0.1)	0 (0.0)	5 (0.1)	

Abbreviations: TUS, thyroid ultrasound.

^a^Kruskal-Wallis *P*-value.

^b^Chi-square *P*-value.

^c^Clinic site identification by letter (site A, B, C, D) for confidentiality purposes: preventing impact on future patient trust in care locations.

**Figure 1.**
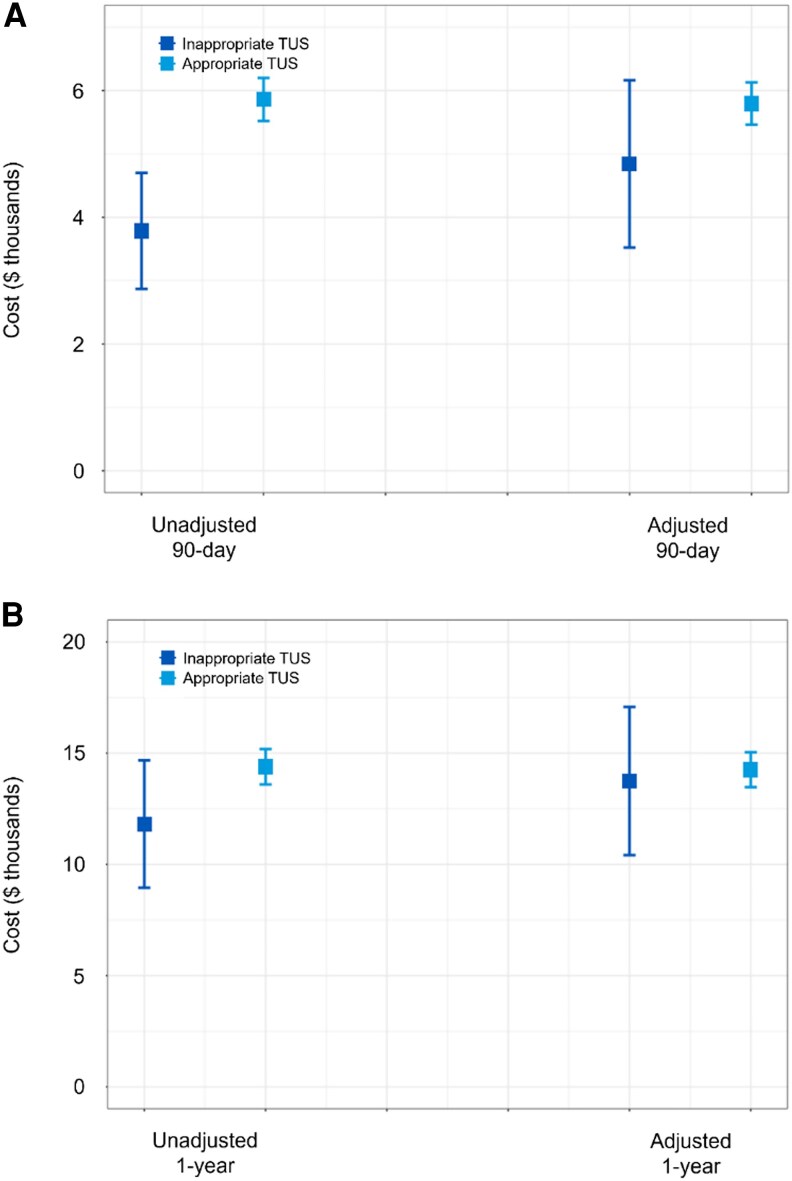
Unadjusted and adjusted all-cause costs. (A) shows costs at 90 days, and (B) shows costs at 1 year following a thyroid ultrasound. Graphs include median (solid square) and 95% range (lines).

Unadjusted total healthcare costs following inappropriate TUS were lower compared to appropriate TUS at both 90 days ($3785 [95% confidence interval (CI): 2869-4701] vs $5859 [95% CI: 5520-6198]) and 1 year ($11 816 [95% CI: 8953-14 678] vs $14 390 [95% CI: 13 594-15 186]) (Fig. 1). However, after adjustment for demographic and clinical covariates, the costs between groups were similar. Adjusted 90-day costs were $4842 [95% CI: 3522-6162] for inappropriate TUS and $5794 [95% CI: 5460-6129] for appropriate TUS; adjusted 1-year costs were $13 748 [95% CI: 10 416-17 080] vs $14 257 [95% CI: 13 473-15 041], respectively.

**Figure 2.**
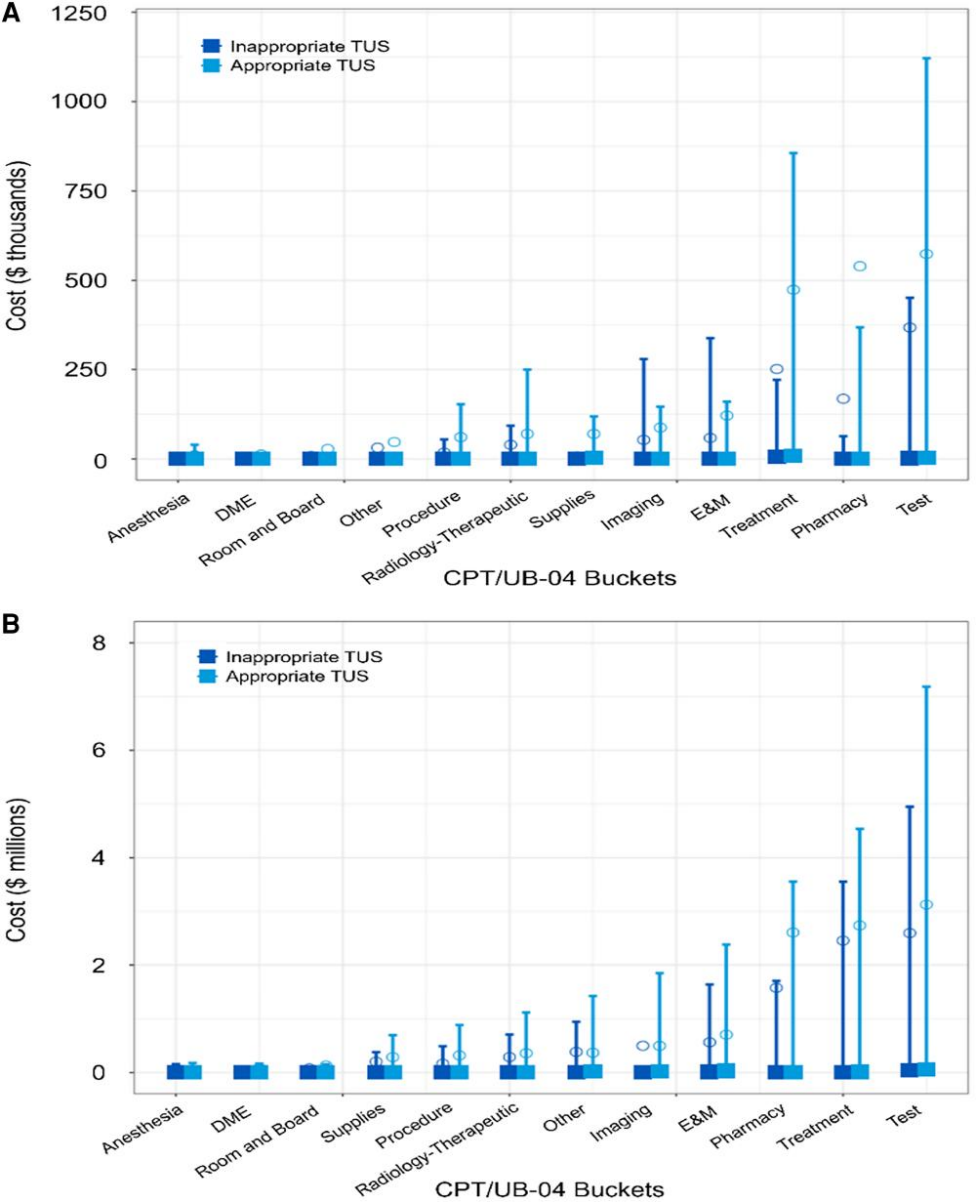
All-cause costs by cost bucket. (A) shows costs at 90 days, and (B) shows costs at 1 year following a thyroid ultrasound. Graphs include mean (solid square) and 95% confidence interval (lines). Abbreviations: CPT, Current Procedural Terminology; DME, durable medical equipment; E&M, evaluation and management; TUS, thyroid ultrasound; UB, Uniform Billing.

Across both timeframes, the largest contributors to total costs in our study population were tests, pharmacy expenses, and treatments (Fig. 2). The total 1-year all-cause healthcare expenditure for the 546 patients who received an inappropriate TUS was $13 748 per patient, totaling approximately $7.5 million for the cohort. While these costs are not directly attributable to the TUS alone, they represent a significant expenditure initiated by a low-value test. To provide a precise and conservative estimate of directly avoidable costs, we calculated the direct procedural costs for the initial diagnostic cascade (Table 2). Based on the estimated number of downstream procedures in our cohort, the directly attributable procedural cost is approximately $576 134. This figure, derived from published payer-negotiated price data and cost-effectiveness analyses [14-16], represents a significant underestimation of the true direct costs, as it excludes other related expenses such as laboratory tests, medications, and additional consultations.

**Table 2.** Estimated direct procedural costs of the initial inappropriate diagnostic cascade based on 2024 Medicare reimbursement rates

Procedure	Cost estimation basis	Estimated cost (per procedure)	Estimated number in the inappropriate thyroid US cohort (n = 546)*^a^*	Estimated direct avoidable procedural costs
Thyroid US	Payer-negotiated price analysis [16]	$395	546	$215 670
FNA with US guidance	Payer-negotiated price analysis [16]	$413	56	$23 128
Partial thyroidectomy	Cost-effectiveness analysis [14]	$15 064	9	$135 576
Total thyroidectomy	Median insurance reimbursement [15]	$15 520	13	$201 760
Total estimated direct costs				$576 134

Abbreviations: FNA, fine-needle aspiration; US, ultrasound.

^a^Number of procedures estimated by applying the rates of downstream procedures from the parent cohort to the current study's cohort of 546 patients with inappropriate thyroid ultrasound.

## Discussion

Thyroid nodules and cancer impose a significant annual burden on the healthcare system, with thyroid cancer alone accounting for an estimated $1.5 billion in costs, which are expected to rise. A substantial portion of these costs could be deemed unnecessary due to the overdiagnosis of many thyroid cancer cases. Previous studies have suggested that many of these overdiagnosed cases result from the inappropriate use of thyroid ultrasound, yet until now, the economic impact of this non-evidence-based practice has undergone limited evaluation.

Leveraging artificial intelligence methods and electronic health record data [10, 18, 19], we found that healthcare costs within 1 year of the initial ultrasound average approximately $14 000. This estimate includes all medical services, not just those directly tied to thyroid care. Although unadjusted 90-day and 1-year healthcare costs were lower in patients who received inappropriate TUS, adjusted analyses revealed that total costs were ultimately similar between groups. This finding indicates that even when initiated with a potentially unnecessary imaging study, the subsequent clinical trajectory, including further testing, prescriptions, and treatments, may mirror that of patients who had a justifiable indication for TUS.

In essence, inappropriate TUS may serve as a gateway to a cascade of additional services, which could explain the convergence of total costs over time. An alternative, and equally plausible, interpretation is that patients who are high utilizers of healthcare are more likely to receive an inappropriate TUS in the first place. In this view, the subsequent high costs are not a direct consequence of the TUS but a reflection of the patient's underlying tendency for high healthcare consumption. We believe these interpretations are not mutually exclusive. Our data suggests that the inappropriate TUS acts as an entry point that activates this pattern of high utilization, committing the patient to a year-long clinical trajectory with costs that ultimately converge with those of patients who had a guideline-concordant reason for imaging. Indeed, this cascade is not trivial; applying the rates of downstream events from the larger parent cohort to our study population suggests that the 546 inappropriate ultrasounds in our cohort would lead to an estimated 56 fine-needle aspirations, 22 thyroidectomies, and 10 new cancer diagnoses. Thus, the problem of low-value care extends beyond the cost of a single test; it involves unnecessarily enrolling a patient, who may already be a high utilizer, into a year of intensified, generalized healthcare consumption with significant clinical and financial consequences.

The largest drivers of healthcare expenditures in our cohort were follow-up tests, pharmacy costs, and treatment procedures. This finding supports the concept of a “cascade of care,” where an initial low-value test triggers substantial downstream consequences, not only in financial terms but also in patient burden. While our direct cost analysis provides a conservative floor for savings, the all-cause cost data suggest that the total economic impact is much larger. When considering that other studies report even higher rates of inappropriate imaging nationally, the cumulative financial implications are likely considerable.

A systematic review of observational studies suggests that the frequency of inappropriate TUS is higher than found in our study [8], with rates as high as 40% compared to our 8%. This discrepancy may be attributed to varying criteria for inappropriateness, different practice cultures, and studied populations. However, this indicates that the frequency of inappropriate use may be higher than reported here, potentially amplifying the healthcare cost impact of inappropriate TUS use.

Addressing inappropriate TUS orders is essential for optimizing resource utilization and preventing unnecessary diagnostic cascades. Interventions to reduce low-value imaging should include provider education on evidence-based guidelines, improved clinical decision-support tools, and strategies to manage patient expectations. Although not the primary focus of this study, observed differences in baseline characteristics between patients receiving appropriate vs inappropriate TUS suggest that certain populations may be at higher risk for unnecessary testing and could benefit from more targeted interventions. For example, patients who underwent inappropriate TUS were more likely to have comorbid thyroid conditions—such as hypothyroidism or Graves' disease—where imaging typically offers limited clinical value unless there is a palpable nodule or other concerning features. These findings highlight clear opportunities for intervention. Additionally, incorporating NLP algorithms, such as the one used in this study, into electronic health records could provide a scalable solution to assess TUS appropriateness in real time, thereby supporting more consistent adherence to clinical guidelines and improving the overall value of care.

This study has several limitations. First, its findings are derived from a single healthcare system, which limits generalizability. Second, although the NLP algorithm demonstrated high accuracy, potential misclassifications of TUS appropriateness may have introduced bias. Third, the cost estimates based on Medicare reimbursement rates may not accurately reflect the expenses incurred by private insurers or patients. Fourth, as this was a retrospective analysis of routine clinical practice, we did not implement a specific standardization protocol for the sonologists or perform a secondary analysis to measure interobserver error among radiologists as part of this research design. The study relied on the final, official reports as documented in the electronic health record by the radiologists. Lastly, our study's primary economic endpoint is all-cause healthcare cost, not costs directly attributable to the TUS. This methodological choice was deliberate. Estimating disease-specific costs is challenging because patients may receive unrelated services or procedures on the same days they get thyroid-related care, making cost attribution difficult. Using all-cause costs captures the total economic activity surrounding a patient after the index event. However, we acknowledge this is a major limitation. As such, these estimates likely include expenses unrelated to the TUS, such as ongoing management of comorbid conditions or unrelated diagnostic evaluations, and may overstate the specific economic impact of inappropriate imaging itself. This is further complicated by the potential for confounding, where patients prone to high healthcare utilization may also be more likely to receive inappropriate tests. Our study cannot disentangle the costs of the TUS cascade from the costs associated with a high-utilizer phenotype. To provide a more conservative estimate, we have presented a separate analysis of direct procedural costs. It is important to note that this direct cost analysis is itself a significant underestimation, as it only includes major procedures and excludes other related expenses such as laboratory tests, medications, and additional consultations. Future studies using matched cohort designs, time-series analyses, or cost-attribution models could provide more precise estimates by isolating services directly triggered by inappropriate imaging.

The adjusted 1-year all-cause healthcare cost for patients undergoing inappropriate TUS was not significantly different from that of patients who received appropriate TUS. This finding suggests that an initial low-value TUS can act as a gateway, committing patients to a year-long trajectory of high healthcare utilization that mirrors the costs of appropriate care. Therefore, inappropriate TUS represents a critical target for improving resource use, not merely to avoid the cost of a single test but to prevent the initiation of a prolonged and expensive care cascade. The significant economic impact detailed in our analysis underscores the importance of implementing targeted interventions to reduce low-value imaging. As we have detailed elsewhere [9], these strategies include specialty-specific provider education and the integration of real-time clinical decision support tools into the electronic health record.

## Data Availability

Restrictions apply to the availability of some or all data generated or analyzed during this study to preserve patient confidentiality or because they were used under license. The corresponding author will on request detail the restrictions and any conditions under which access to some data may be provided.

## References

[1] Genere N, El Kawkgi OM, Giblon RE, et al Incidence of clinically relevant thyroid cancers remains stable for almost a century: a population-based study. Mayo Clin Proc. 2021;96(11):2823‐2830. 34736609 10.1016/j.mayocp.2021.04.028 PMC9645772

[2] Howlader N, Noone AM, Krapcho M, et al *SEER cancer statistics review, 1975-2018*. National Cancer Institute; 2021.

[3] Ramsey S, Blough D, Kirchhoff A, et al Washington State cancer patients found to be at greater risk for bankruptcy than people without a cancer diagnosis. Health Aff (Millwood). 2013;32(6):1143‐1152. 23676531 10.1377/hlthaff.2012.1263 PMC4240626

[4] Lubitz CC, Kong CY, McMahon PM, et al Annual financial impact of well-differentiated thyroid cancer care in the United States. Cancer. 2014;120(9):1345‐1352. 24481684 10.1002/cncr.28562 PMC3999178

[5] Barrows CE, Belle JM, Fleishman A, Lubitz CC, James BC. Financial burden of thyroid cancer in the United States: an estimate of economic and psychological hardship among thyroid cancer survivors. Surgery. 2020;167(2):378‐384. 31653488 10.1016/j.surg.2019.09.010

[6] Haymart MR, Banerjee M, Reyes-Gastelum D, Caoili E, Norton EC. Thyroid ultrasound and the increase in diagnosis of low-risk thyroid cancer. J Clin Endocrinol Metab. 2019;104(3):785‐792. 30329071 10.1210/jc.2018-01933 PMC6456891

[7] Soto Jacome C, Segura Torres D, Fan JW, et al Drivers of thyroid ultrasound use: a retrospective observational study. Endocr Pract. 2023;29(12):948‐954. 37722595 10.1016/j.eprac.2023.09.006 PMC10843084

[8] Edwards MK, Iñiguez-Ariza NM, Singh Ospina N, Lincango-Naranjo E, Maraka S, Brito JP. Inappropriate use of thyroid ultrasound: a systematic review and meta-analysis. Endocrine. 2021;74(2):263‐269. 34379311 10.1007/s12020-021-02820-z PMC10292117

[9] Larios F, Toro-Tobon D, Jacome CS, et al Factors and outcomes of inappropriate thyroid ultrasonography. JAMA Otolaryngol Head Neck Surg. Published online August 7, 2025. Doi: 10.1001/jamaoto.2025.2049 PMC12332758 40773204

[10] Soto Jacome C, Segura Torres D, Fan JW, et al Thyroid ultrasound appropriateness identification through natural language processing of electronic health records. Mayo Clin Proc Digit Health. 2024;2(1):67‐74. 38501072 10.1016/j.mcpdig.2024.01.001 PMC10947349

[11] Toro Tobon D, Thao V, Borah B, et al 2025. Supplement 1. Criteria for appropriate thyroid ultrasound characterization. Figshare.

[12] Visscher SL, Naessens JM, Yawn BP, Reinalda MS, Anderson SS, Borah BJ. Developing a standardized healthcare cost data warehouse. BMC Health Serv Res. 2017;17(1):396. 28606088 10.1186/s12913-017-2327-8 PMC5469019

[13] Toro Tobon D, Thao V, Borah B, et al Supplement 2. Cost data categorization and buckets creation. Figshare. 2025.

[14] Shrime MG, Goldstein DP, Seaberg RM, et al Cost-effective management of low-risk papillary thyroid carcinoma. Arch Otolaryngol Head Neck Surg. 2007;133(12):1245‐1253. 18086967 10.1001/archotol.133.12.1245

[15] Zheng F, Huang Y, Wright J, Kuo JH. Out-of-pocket costs for patients undergoing thyroid surgery. Ann Surg. 2022;276(6):e937‐e943. 34261887 10.1097/SLA.0000000000005078

[16] Xiao R, Rathi VK, Gross CP, Ross JS, Sethi RKV. Payer-negotiated prices in the diagnosis and management of thyroid cancer in 2021. JAMA. 2021;326(2):184‐185. 34086052 10.1001/jama.2021.8535 PMC8278265

[17] Manning WG, Basu A, Mullahy J. Generalized modeling approaches to risk adjustment of skewed outcomes data. J Health Econ. 2005;24(3):465‐488. 15811539 10.1016/j.jhealeco.2004.09.011

[18] Toro-Tobon D, Loor-Torres R, Duran M, et al Artificial intelligence in thyroidology: a narrative review of the current applications, associated challenges, and future directions. Thyroid. 2023;33(8):903‐917. 37279303 10.1089/thy.2023.0132 PMC10440669

[19] Loor-Torres R, Duran M, Toro-Tobon D, et al A systematic review of natural language processing methods and applications in thyroidology. Mayo Clin Proc Digit Health. 2024;2(2):270‐279. 38938930 10.1016/j.mcpdig.2024.03.007 PMC11210322

